# Ionospheric perturbation during the South American total solar eclipse on 14th December 2020 revealed with the Chilean GPS eyeball

**DOI:** 10.1038/s41598-021-98727-w

**Published:** 2021-10-13

**Authors:** Mahesh N. Shrivastava, Ajeet Kumar Maurya, Kondapalli Niranjan Kumar

**Affiliations:** 1 Universidad Católica del Norte, Antofagasta, Chile; 2 Department of Physics, Doon University, Dehradun, India; 3 National Centre for Medium Range Weather Forecasting, Ministry of Earth Sciences, Noida, India

**Keywords:** Atmospheric dynamics, Solar physics

## Abstract

The influence of the South American total solar eclipse of 14th December 2020 on the ionosphere is studied by using the continuous Chilean Global Positioning System (GPS) sites across the totality path. The totality path with eclipse magnitude 1.012 passed through the Villarrica (Lon. 72.2308°W and Lat. 39.2820°S) in south Chile during 14:41:02.0 UTC to 17:30:58.1 UTC and maximum occurred ~ 16:03:49.5 UTC around the local noon. The vertical total electron content (VTEC) derived by GPS sites across the totality path for two PRN’s 29 and 31 show almost 20–40% of reduction with reference to ambient values. The percentage reduction was maximum close to totality site and decreases smoothly on both sides of totality sites. Interestingly, the atmospheric gravity waves (AGWs) with a period ~ 30–60 min obtained using wavelet analysis of VTEC timeseries show the presence of strong AGWs at the GPS sites located north of the totality line. But the AGWs do not show any significant effect on the VTEC values to these sites. Our analysis suggests, possibly an interplay between variability in the background plasma density and eclipse-generated AGWs induced plasma density perturbation could explain the observations.

## Introduction

Solar eclipses always fascinated mankind and remained pivotal for many groundbreaking discoveries, thus still attracting the scientific community to look for discoveries associated with eclipses. The ionospheric effects of solar eclipse are now a widely studied phenomenon as evident with several publications^1–6^. Further, it is also well known that the solar eclipse effect differs in terms of eclipse location, seasons, region of the atmosphere, occurrence time, duration, types (partial, annular, and total). Thus, it remained attractive for the radio/ionospheric science community.

A solar eclipse provides an opportunity to study the response of the thermosphere/ionosphere due to the sudden decrease in solar ionizing radiation towards the nighttime values due to the complete blockage of the solar disk by the moon. Thus, the initial focus was to study photoionization and loss process in the ionosphere due to eclipse^7,8^. After the detailed description of atmospheric gravity waves (AGWs) by Hines^9^ and subsequent work by Chimonas and Hines^2^, which suggested solar eclipses can be the sources of AGWs. The detailed theory proposed by Chimonas and Hines^2^ is based on the supersonic speed of the lunar shadow that sweeps across the Earth and creates an excellent spot in the atmosphere. Such fast-moving shadow creates strong gradients in temperature and ionization flux and generates a non-equilibrium state in the atmosphere in the form of AGWs. Further, another theory proposed by Fritts and Luo^10^, suggested the eclipse-induced ozone heating created perturbations in the stratosphere, which may propagate upwards and induce disturbances into the thermosphere–ionosphere system. Further subsequent works suggested many different mechanisms for wave generation and enhancement at ionospheric altitude during the eclipse^11^, also the local conditions such as local time, background wind^12^, topography might affect AGWs propagation^13^ and can cause significant variation in the ionospheric electron density during an eclipse. However, inconclusive results of the solar eclipse observations rise from the fact that different solar eclipses produce different plasma motions. Indeed, the travel cone geometry and its angular effects on the magnetized plasmas are different for each eclipse.

With the recent advancement in radio remote sensing techniques, it is now possible to continuously monitor solar eclipse effects on the ionosphere over a large geographical region. The GPS derived total electron content is one of the best widely used cost-effective tools for continuous monitoring of ionospheric changes during solar eclipse^14–18^. When GPS signals (pseudorange and carrier phase) travel through the ionosphere, it gets delayed due to its dispersive properties' by interacting with plasma. The propagation delay of the GPS signal in the ionosphere is directly proportional to TEC. Several authors have reported a decrease in TEC associated with the solar eclipse^15,17,19^. Jonah et al.^19^ studied 2nd July 2019 South American, total solar eclipse effect in the ionosphere using GPS TEC signal and have reported ~ 35% TEC suppression along the path of totality and south of the equatorial anomaly region. Afraimovich et al.^15^ have also analyzed GPS-derived TEC for the 09th March 1997 total solar eclipse and observed ~ 1–3 TEC unit decrease in TEC. Kumar Vijay et al.^17^ analyzed GPS TEC data for the 22nd July 2009 total solar eclipse and reported ~ 43% decrease in electron density during maximum eclipse. Apart from the TEC variations during the eclipse, several recent works have used GPS derived TEC signal to study eclipse variations along and across its totality path and have reported the generation of AGWs during the solar eclipse which affecting TEC variations in the ionosphere^9,12,18,20^.

The recent work by Maurya et al.^12^ about Total solar eclipse 2019 (from here onwards referred to as TSE2019) demonstrated interesting features of AGWs generated during 2nd July 2019 total solar eclipse occurred in the south American region. They have analyzed TEC signals from 24 GPS sites located across the totality path and observed a decrease in TEC on the eclipse day compare to the unperturbed day for GPS sites located south of the totality path, whereas an increase in TEC for the GPS sites located north of totality path. They have suggested an increase in TEC during the eclipse for the GPS sites located north of totality could be due to an interplay between direct eclipse effect on the ionosphere plasma density and eclipse generated AGWs induced plasma density perturbation, which was supported by the presence of strong AGWs generated due to the solar eclipse over these GPS sites.

The 14th December 2020 total solar eclipse (from here onward referred to as TSE2020) occurred in the South American region. The maximum eclipse magnitude was reported ~ 1.0254 with a maximum totality ~ 2 min and 10 s visible in Argentina. The solar eclipse's totality was visible from the South Pacific Ocean, southern South America, and the South Atlantic region. The partial eclipse was witnessed in much wider regions covering countries like Chile, Peru Bolivia, Ecuador Paraguay Uruguay, and Brazil. More information about this eclipse can be found at (https://eclipse.gsfc.nasa.gov/SEgoogle/SEgoogle2001/SE2020Dec14Tgoogle.html). Over Chile, totality made landfall in the Puerto Saavedra (Lon. 73.3987°W and Lat. 38.7837°S) and traverses through portions of the Araucanía and Los Ríos regions. The total solar eclipse duration in Chile was from 11:59:57.4 CLT to 12:01:59.9 CLT Chilean local Time (CLT = UTC—4:00:00) moving from South-East towards North-West. However, the partial eclipse duration was much longer varied from 10:38:32.2 CLT to 13:28:18.6 CLT around 2 h 49 min 46.4 s. For reference, the sunrise and sunset time on 14th December 2020 in the city of Temuco (was in totality path) in Chile was ~ 06:19 CLT and 21:11 CLT respectively. Thus, the eclipse happened during the forenoon to early afternoon time with maximum eclipse at the noon time over the Chilean region. The major objective of this work is to understand the important role of eclipse generated AGWs in modifying ionospheric electron density. The results of TSE2019 show the increas in TEC values for the site located north of the totality line owing to the interplay between the direct eclipse effect and eclipse generated AGWs. We have chosen similar configurations of GPS sites from the Chilean GPS eyeball network located on both sides (north and south) of the totality path and have assessed the background conditions to better understand AGWs propagation.

## Methodology: estimation of TEC from GPS data

**Figure 1:**
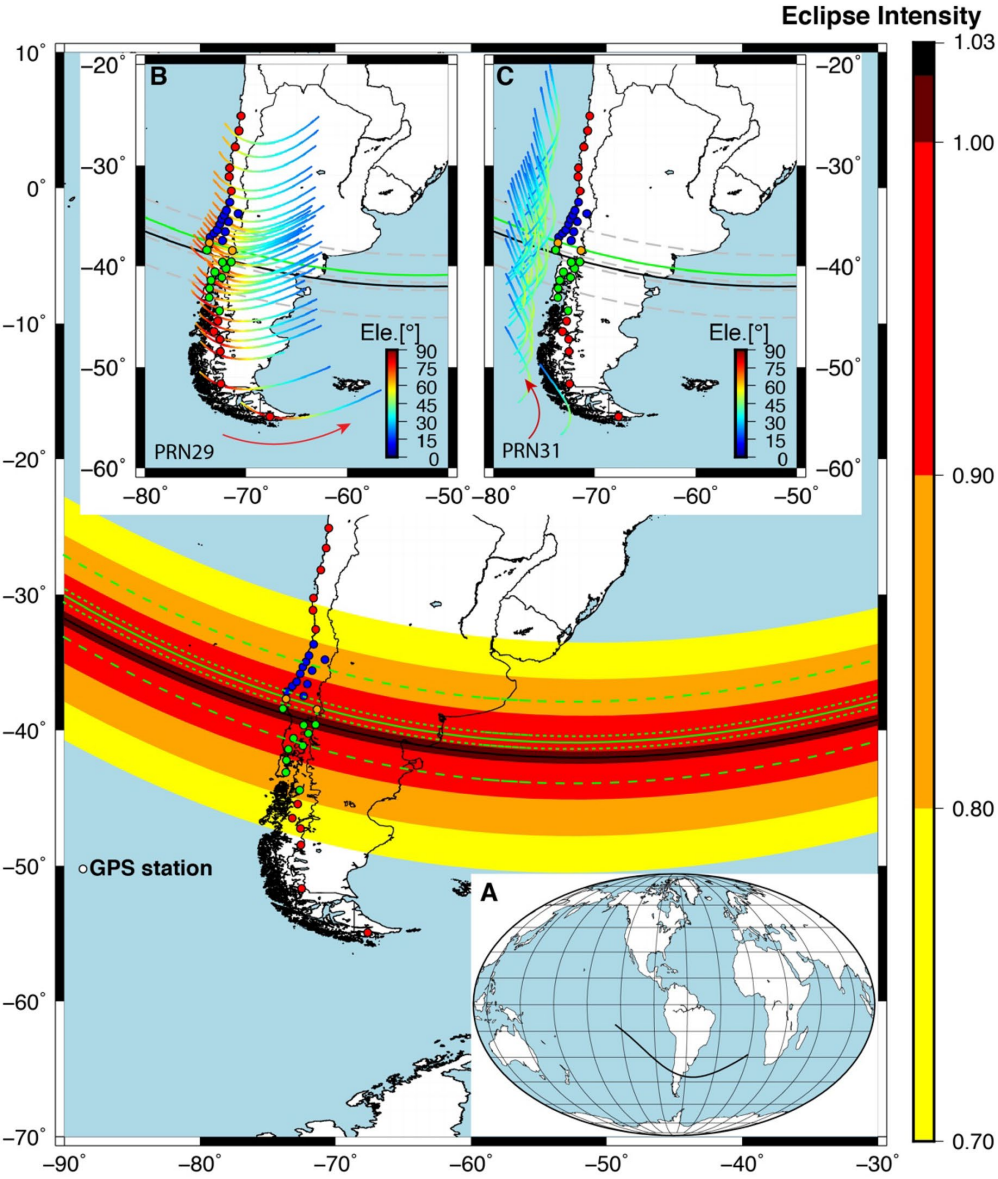
Geographic map of Chile and the total solar eclipse on Dec 14, 2020. The color dots show the GPS sites for TEC analysis. The orange color dots represent totality path at altitude 350 km for the TEC analysis. The blue dots represent the north GPS site of the totality and green color dots south of the totality. The red dots show GPS sites away from the 80% totality region. Inset (**A**) the path of the total solar eclipse central line on the world map. (**B**) The elevation of GPS PRN29 path at 350 km altitude with respect to ground GPS sites with color table from blue to red and IPP represent on the path with color circles. (**C**). The elevation of GPS PRN31 path at 350 km altitude with respect to ground GPS sites with color table from blue to red and IPP represent on the path with color circles. The Figure is prepared using the GMT 5.4.5^37^.

The TSE2020 details are shown in Fig. 1 and its subsections (A, B and C). The eclipse conditions at the ground levels are shown in Fig. 1 as colour-coded with 100% up to 70% eclipse obscuration. The central line of the eclipse totality path at ground level is shown in black colour. The global path of the central totality line at ground level is shown in Fig. 1A. The path of the totality at altitude 350 km is shown with a continuous green line in Fig. 1A. The global path of the solar eclipse was from the South Pacific ocean, southern South America, and the South Atlantic region. We have used a total of 24 GPS sites, which lies in the totality as well as up to ~ 80% solar obscuration on both sides of the totality line. For the comparison with results of TSE2019, and to validate any enhancement of TEC in case TSE2020, we analyzed 12 additional GPS sites, 6 GPS sites located north, and 6 GPS sites located south of totality. These results are provided in the supplementary information (see Figs. S1, S2, S3, and Table S1), thus covering the solar obscuration up to ~ 50% on both sides of totality. There were 2 GPS sites PECL and LNQM on the path of the total solar eclipse at ionospheric pierce point (IPP) at altitude 350 km, shown with orange color-filled circles (in Fig. 1 and Table 1), 10 sites (IMCH, CRRH, PGLL, CURR, OSOR, PTRO, MUER, LNCM, QLLN, and TPYU) south of totality shown with green colour filled circles and 12 GPS sites (RCSD, PCMU, SBLL, ILOC, CONS, SJAV, PELL, VITA, CLL1, HLPN, PLVP, and UDEC) north of totality shown with blue colour filled circles. The 12 GPS sites (six sites from each side of totality lines) shown in red-coloured filled circles are provided as supplementary information (Fig S3). The details about the GPS sites and eclipse conditions at the ground level can be found in Table 1. Further, as the solar eclipse effect changes with altitude and GPS TEC provide major contribution from F region ~ 350 km altitude, thus we have also estimated eclipse conditions at the ionospheric height of 350 km by the method suggested by Verhulst et al.^21^ and shown with green lines (totality central line as solid green colour lines, totality north, south limit and 90% obscuration lines as dashed green colour lines).

**Table 1:**
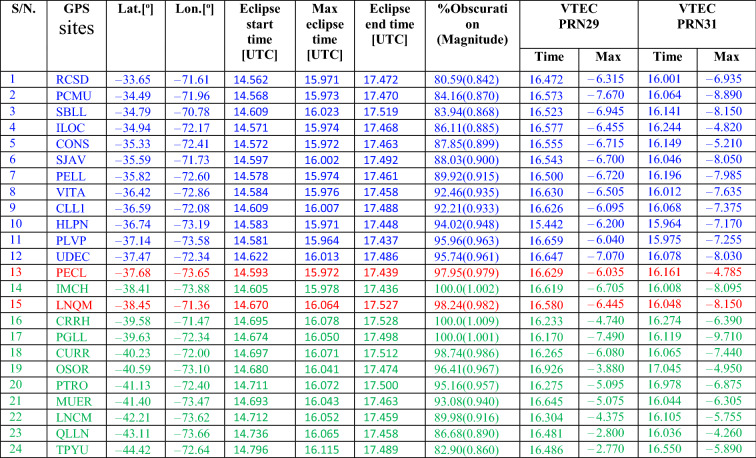
Detail about the GPS sites analyzed, their location, eclipse condition, magnitude and change in VTEC at each site.

The GPS data used in the current work are from continuously operating and managed by the Chilean Centro Sismológico Nacional (CSN). The GPS receivers collect dual-frequency signals operated at L1 (1575.42 MHz) and L2 (1227.60 MHz) with different sampling rates. The data obtained from each receiver are in the Receiver Independent Exchange Format (RINEX) and processed for TEC format with uniformly 30 s sampling rate for all sites. The format conversion was carried out by using GPS_GOPI software (http://seemala.blogspot.com/2017/09/gps-tec-program-ver-295.html) with utilization of Differential Code Baises (DCBs) files (from ftp://ftp.aiub.unibe.ch/CODE/), Rinex observation files for elevation and azimuth angles of the satellites is used to obtain the TEC parameters^22,23^. The VTEC is estimated from the line-of-sight TEC (STEC) values using a mapping function and is associated to an IPP latitude and longitude, assuming the ionosphere to be compressed into a thin shell Single layer Ionosphere Model (SLIM) at the peak ionospheric height of 350 km^24^.

The GPS TEC data recorded on eclipse day (14th December 2020) and before and after the eclipse day (13th, 15th December 2020) is from 14:00:00 UTC until 18:00:00 UTC is used in this work. The data were filtered to include only signals from all satellites with elevation angle > 20^ο^ to avoid multipath disturbances, noise caused by high-rise buildings or tall trees, etc. The two pseudo-random numbers (PRNs). PRN29 and PRN31 have been chosen based on their paths, elevation angle and duration suitable for eclipse analysis. It is important to have a satellite covering a longer time before and after the eclipse and in the high elevation range to avoid the multipath and other signal diffraction phenomenon. The path details of PRN29 and PRN31 at IPPs are color-coded with reference to elevation angle (in degree) and shown in Fig. 1B and C respectively. The red arrows in both Figs. (1B and 1C) show satellite propagation directions.

## Results of VTEC analysis

**Figure 2:**
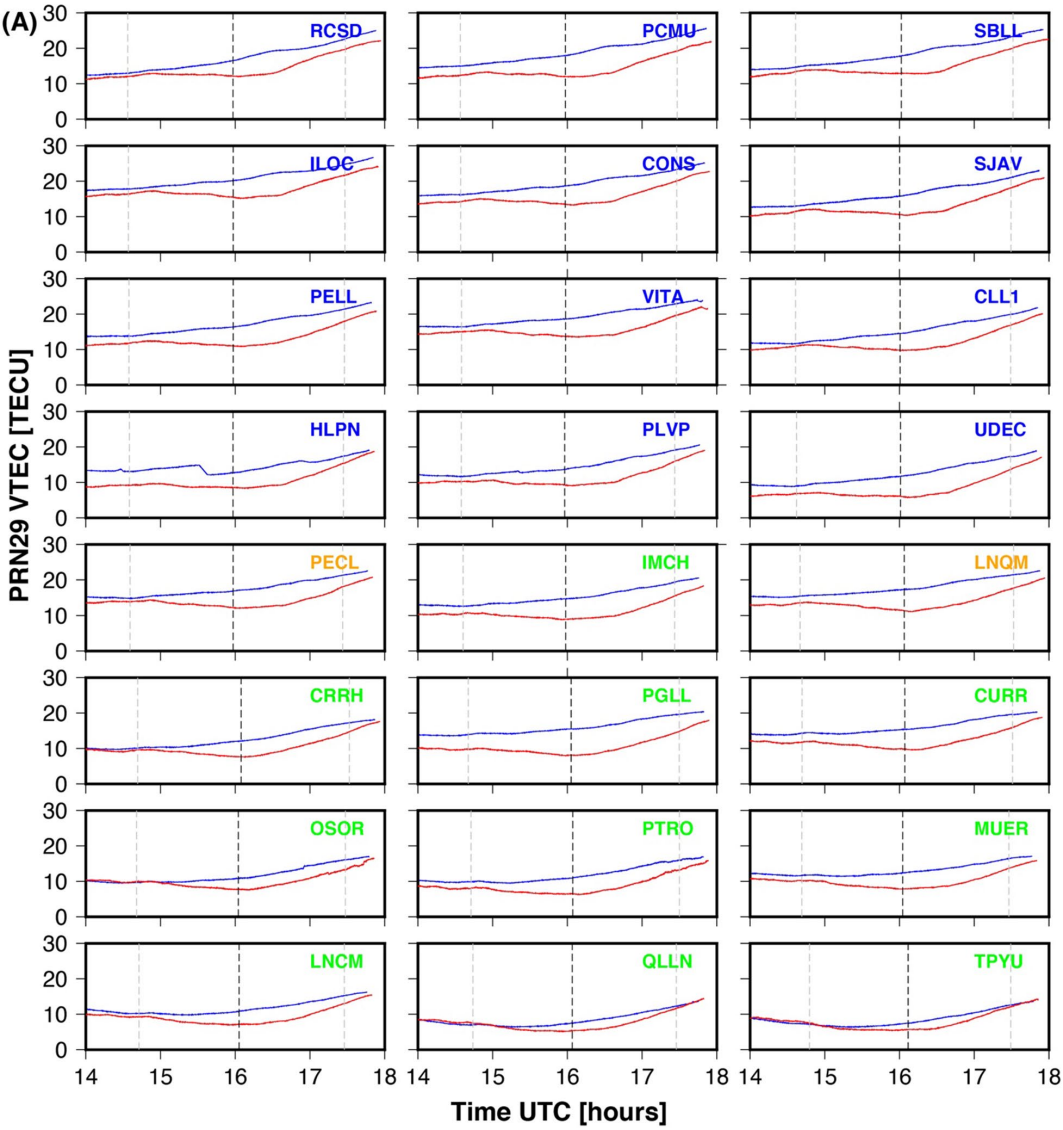

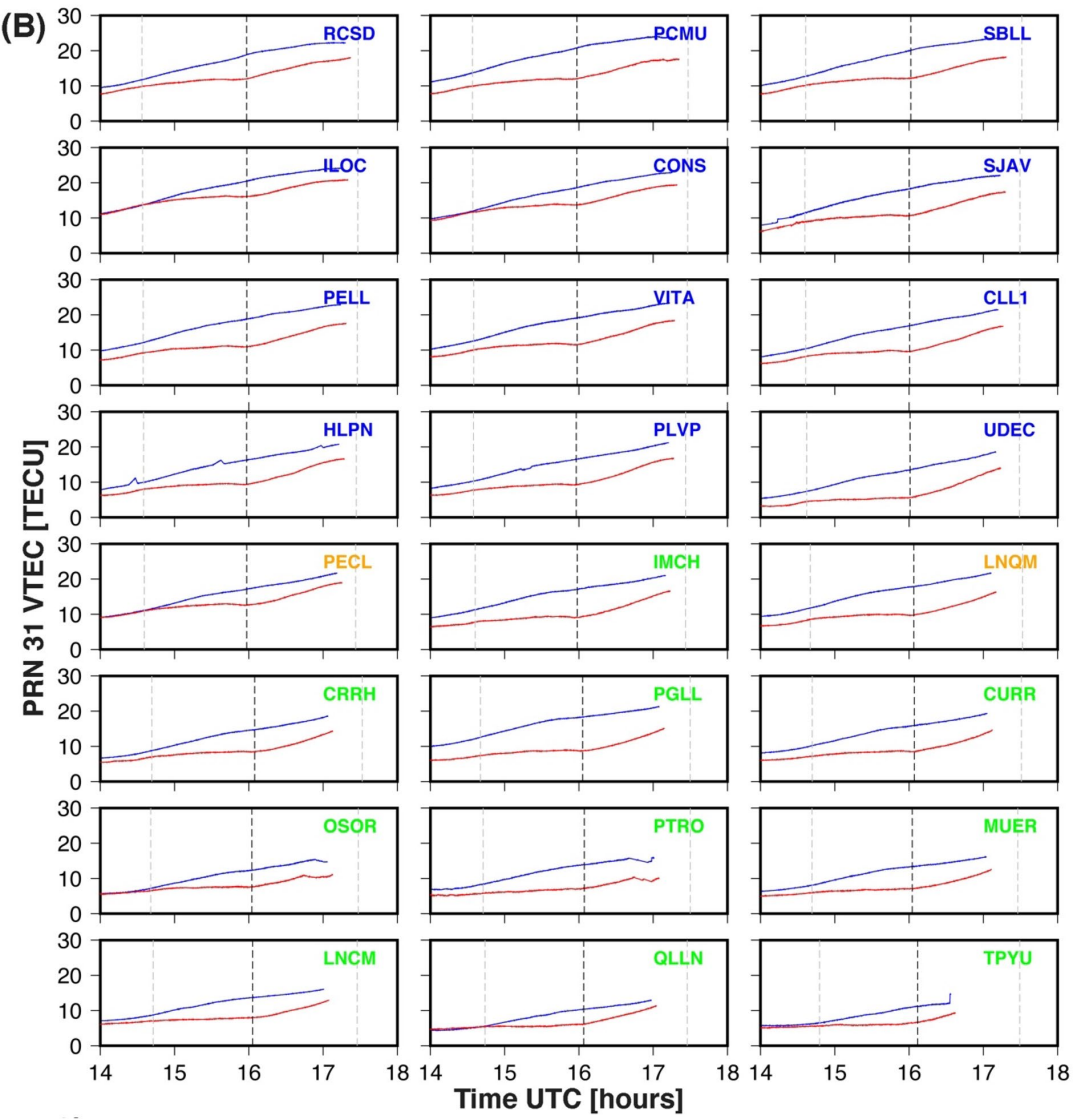
(**A**) The VTEC plots of the PRN29. The red color line shows the average VTEC before and after the total solar eclipse day as DOY 348 and 350. The blue color line shows the VTEC on the Total solar eclipse day as DOY 349. The down subplot shows the reduction of the VTEC of GPS sites from north to south. (**B**) The VTEC plots of the PRN31. The red color line shows the average VTEC before and after the total solar eclipse day as DOY 348 and 350. The blue color line shows the VTEC on the Total solar eclipse day as DOY 349. The down subplot shows the reduction of the VTEC of GPS sites from north to south. The plots are arranged from bottom (South: GPS sites name in green color) to the top (North: GPS site named with blue color). The GPS sites in totality region named with orange color. Eclipse magnitude at each site added at the upper left corner. The grey dotted vertical line is showing maximum eclipse time at each site. The The Figure is prepared using the GMT 5.4.5^37^.

Three days of VTEC data for each GPS site is utilized in this work. The three days include eclipse day (14th December 2020), the day before the eclipse (13th December 2020), and the day after the eclipse (15th December 2020). The results for both PRNs (29 & 31) are presented in Fig. 2A and B respectively. Figure 2A and B show the VTEC variation as a function of time for the mean (blue line, estimated using two days (13th & 15th December 2020, VTEC data) and eclipse day (red line) and plotted for each GPS site. Each plot is shown for 4 h duration (i.e., 14–18 UTC) which corresponds to the eclipse duration over the region. The dashed grey vertical line on each subplot shows the maximum eclipse time and dim grey vertical dotted lines show the initial and ending of the solar eclipse. The GPS sites in each subplot of Fig. 2A and B are colour coded as per the eclipse obscuration percentage at the altitude of 350 km. The totality GPS sites (PECL & LNQM) are shown with orange colour, while GPS sites within < 90% obscuration and located north of totality are shown in blue colour. The GPS sites located south of the totality line and of < 90% obscuration are shown in green.

**Figure 3:**
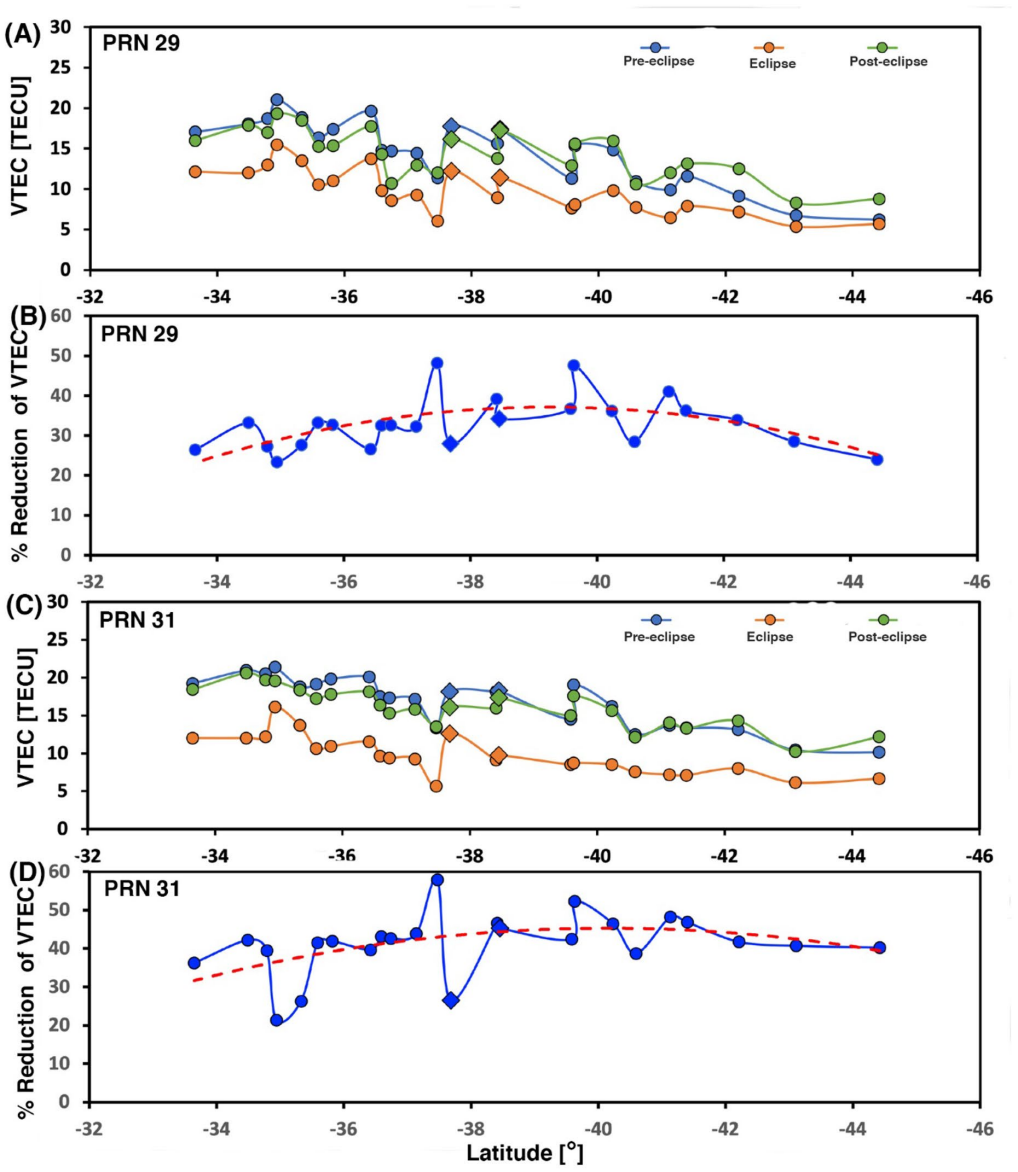
The VTEC of the PRN29 and PRN31 of the GPS sites from the north to south at Total Solar eclipse time, pre-eclipse (DOY, 348), post-eclipse (DOY, 350) and on the eclipse day (DOY, 349) The Figure is prepared using the GMT 5.4.5^37^.

December is the summertime in the southern hemisphere, and the day length is ~ 1.5 h longer. As per Table 1, the approximate eclipse duration at all the GPS sites ~ 14.50 UTC—17.50 UTC, which corresponds to ~ 10.50 CLT—13.50 CLT. This time approximates the local noontime at the Chilean GPS sites, which provides an opportunity to study ionospheric perturbation in the total solar eclipse effect during the peak of ionization. The VTEC time series variation for PRN29, shows a time lag between maximum eclipse time and maximum decrease in VTEC on the eclipse day. The maximum decrease happened on an average of ~ 30 min after the maximum eclipse at the GPS site (Table 1). The time lag for the totality GPS sites ~ 40 min & ~ 35 min respectively for PECL & LNQM. The time lag decreases as one moves both north and south of the totality path, further, the time lag is comparatively lower (~ 20–25 min) for the GPS sites located south of the totality path compared to GPS sites located north of totality (~ 30 min). For PRN31, the time of maximum eclipse and maxima decrease in VTEC almost coincides with few GPS sites. For example, the time lag for totality GPS sites PECL and LNQM estimated at ~ 11 min & 1 min. Thus, present results further verified the delay (time lag), which is very well reported in many previous works^16,17^. To visualize a clear reduction of VTEC at 24 GPS sites, we provided a time series of absolute VTEC in Fig. 3. on three days: eclipse day (DOY, 349), the day before the eclipse (DOY, 348), and the day after the eclipse (DOY, 350) at maximum eclipse time, i.e., mentioned in Table 1 for both PRN29 and PRN31. The TEC values for both PRNs and all three days show a decrease as one moves from north to south. Figure 3 suggests that background TEC values are higher in the north of the totality path compare to the south. On average, north of the eclipse, background TEC varies from 15 to 20 TECU, while in the south it varies from 10–15 TECU for both PRNs. On eclipse day both PRNs show decrees in absolute TEC values (See Fig. 3A and C, brown colour line) as compared to normal day values. Interestingly, absolute TEC values on eclipse day are again higher at the GPS sites north of totality compare to the GPS sites south of totality. The magnitude of VTEC reduction is about 2 ~ 6 TECU and 4 ~ 8 TECU from south to north for PRN29 and PRN31, respectively. Hence, the higher values of TEC on the sites located north of the totality path most probably related to the low latitude of these sites. Further, the percentage decrease in TEC on eclipse day with reference to mean of two days is also estimated for both PRNs and shown in Fig. 3B and D. The trend line for PRN29 passing along the eclipse path shows a maximum decrease at totality sites (~ 35%), while a decrease of ~ 20% on both sides of the totality line. For PRN31 passing perpendicular to the eclipse path a similar trend is observed, but maximum decrease at totality site ~ 40%, while decreases to ~ 30% at both ends. Thus, the TEC decrease for PRN31 is higher than that for PRN29, this is most probably related with the geometry of satellite path and solar eclipse totality path. As mentioned above, for PRN29, satellites path is along the totality path, thus observed almost constant totality effect for longer duration whereas, for PRN31, satellites path is across the totality path, and observe varying totality effect for comparatively short duration. This is also supported by timing of the maximum decrease in TEC and maximum eclipse timing at each site for both PRNs as discussed above.

## Discussion

The South American TSE2020 provides a rare opportunity to investigate the significant role of eclipse generated AGWs on the modification of ionospheric plasma density. The major observational features of TSE2020 effect on the ionosphere: decrease in VTEC on eclipse day compare to mean of two days for the sites located on both sides of totality path for PRNs 29 & 31; VTEC values are higher for the sites north of totality compare to the south of totality for both PRNs even on the eclipse day; the VTEC values on eclipse day for the GPS sites located north of totality show more fluctuations compare to the south of totality; the percentage reduction in VTEC show maximum close to totality site and decrease both sides of totality line; and the magnitude of percentage reduction in VTEC is relatively higher for PRN31 compare to PRN29. At the PECL and LNQM sites which exist in the totality region at 350 km height, there is a reduction of the VTEC during the eclipse ~ 6.0–6.4 TECU and ~ 4.0–8.0 TECU with respect to unperturbed VTEC value for PRN29 and PRN31 respectively.

Before, studying the eclipse effect on the ionosphere, it is essential to identify the space weather (solar flare and geomagnetic storm) conditions which are important contributors of VTEC variability^25,26^. We have confirmed from https://www.spaceweatherlive.com/ and found that there was a small solar flare C4.03 that occurred with maxima at 14:37 UTC on 14th December 2020, as this is C class flare and happened before the eclipse, thus, have no significant effect on the TEC. The geomagnetic data is downloaded from the world data centre Kyoto Japan (http://wdc.kugi.kyoto-u.ac.jp/). We have checked three hourly planetary K index (K_p_) and disturbance storm time index (Dst) providing the conditions for the geomagnetic activity for three days during Dec 13–15, 2020. The Maximum K_p_ index was + 3 on 13th December 2020 during 03 UT. The minimum Dst was -13nT at 07 UTC on 13^th^ December 2020. These values show quiet geomagnetic conditions. Thus, there was no magnetospheric input during the analysis period i.e., Dec 13–15, 2020.

**Figure 4:**
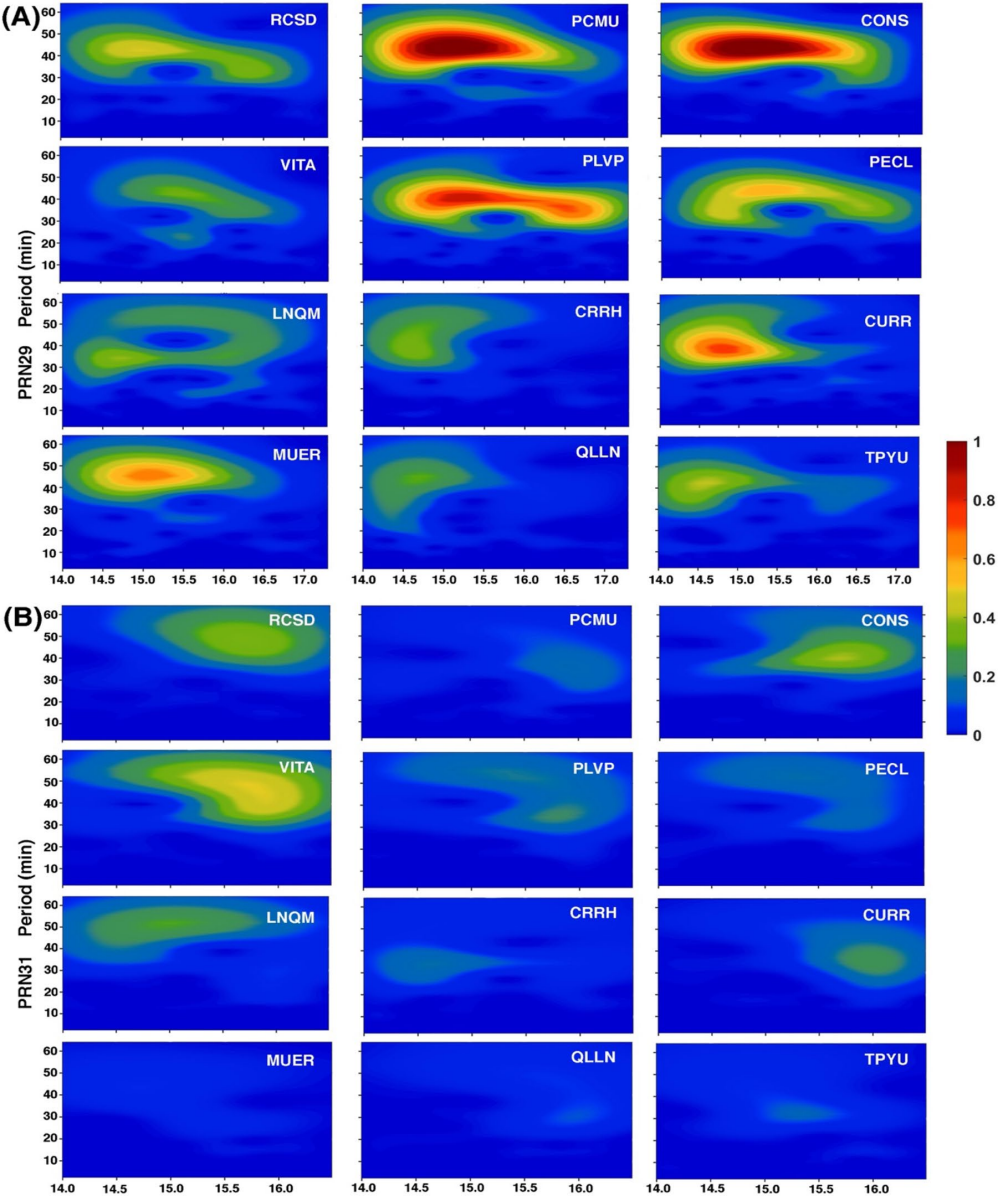
Wavelet analysis of VTEC time series of PRN 29 and PRN31 using Morlet mother wavelet, show atmospheric gravity waves with period ~ 30–60 min are present during eclipse. Six representative GPS sites are shown. (**A**) The wavelet analysis of PRN29 of randomly chosen GPS sites VTEC time series. (**B**) The wavelet analysis of PRN31 of randomly chosen GPS sites VTEC time series. The Figure is prepared using the GMT 5.4.5^37^.

The peculiar features observed during TSE2019 were explained based on the interplay between the eclipse effect on the ionospheric plasma density and eclipse generated AGWs, whose propagation towards northward was supported by background wind. Thus, the results of TSE2019 indicated an important role of eclipse generated AGWs and background wind in affecting ionospheric plasma. In the present work, we are revisiting the role of eclipse induced AGWs and their effect on the ionosphere to explain the observations. To see the presence of AGWs, we have analyzed VTEC data using the Morlet wavelet analysis technique as discussed in many of the previous works^4,27,28^ and also used in Maurya et al.^12^. We performed wavelet analysis for 12 GPS sites for PRN 29 and PRN31. The GPS sites are chosen in such a way to cover the site up to 80% eclipse obscuration. The wavelet analysis results are shown in Fig. 4A and B for PRNs 29 and 31 respectively. For the analysis, first, we have filtered fluctuations greater than 2 h to see the high-frequency fluctuations caused by the eclipse. For the filtered time series, we performed the Morlet wavelet analysis for the site running from south to north. In Fig. 4, wavelet analysis for GPS sites in totality region with reference to the green solid line (PECl, LNQM), partial eclipse region (CRRH, CURR, MUER, QLLN and TYPU < 90% eclipse obscuration), and GPS sites located extreme south and north (RCSD, PCMU CONS, VITA and PLVP < 90% eclipse obscuration) is shown. As one can see in Fig. 4A, for PRN29, moving along the solar eclipse path of GPS sites sense the clear AGWs signature with a period ~ 30–60 min. The AGWs at totality sites are very weak in intensity, while the site away from totality shows strong intensity. This is because the wave amplitude increases away from the source regions. Further, at the site far north and south of the totality line, intensity is again very weak. Furthermore, the intensity of AGWs is relatively strong for the GPS sites located north of the totality line compare to GPS sites south of the totality line. As shown in Fig. 4B for the PRN31, crossing the solar eclipse path of GPS sites showed similar AGWs perturbations at different GPS sites as for PRN29, but with very weak intensity. The AGWs at different GPS sites for both PRNs show similar periods (~ 30–60 min), except the stronger wave intensity for the sites away from totality suggests that these waves generated from the same source, i.e., solar eclipse. As suggested by Somsikov^29^, AGWs generated by the solar eclipse, propagates as a wave, away from the totality path. Further, the amplitude of AGWs is higher for the sites north of totality and also for the PRN29, moving along the eclipse path. Thus, we speculate, it is possible that background conditions supporting AGWs propagation northward and along the eclipse path.

**Figure 5:**
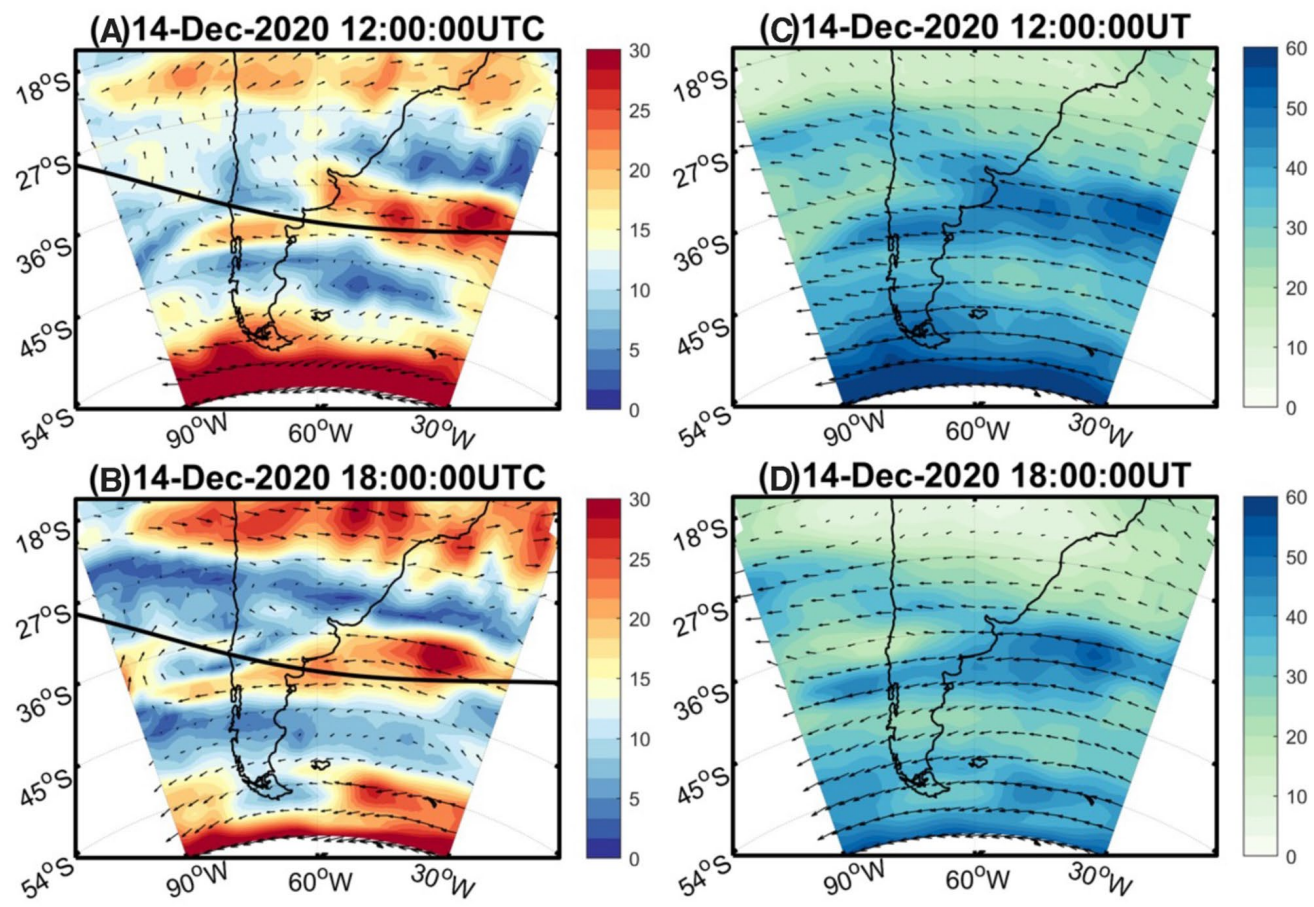
(Left panel) The wind shear (ms^−1^) obtained from the difference between the mesospheric (~ 80 km) and stratospheric winds(~ 40 km) for (**A**) 12 UT and (**B**) 18 UT on 14 December 2020. The eclipse center path at 350 km is also overlayed in (**A**) and (**B**). (right panel) (**C**) and (**D**) Similar to the (**A**) and (**B**) but for mesospheric background winds (~ 80 km). The winds data is obtained from the ERA5 renalysis dataset. The Figure is prepared using the GMT 5.4.5^37^.

As suggested in many previous works^12,30,31^ the background winds and vertically sheared horizontal wind affect AGWs propagation. Observational reports in the upper atmosphere also indicate that the horizontal propagation of AGWs often indicates either horizontal or perpendicular to the background wind direction. The contrasting features noted in the AGWs amplitudes in the north and south (Fig. 4) can be explained by understanding these background conditions and the vertical shear of horizontal winds. Here, we first showed the vertical shear of background wind associated with mesosphere and stratosphere (Fig. 5A,B) on the eclipse day of 14th December 2020. Previous studies of eclipse generated AGWs indicate the source might also be in the stratosphere^2–4^. Hence, it is important to understand the changes in the direction of horizontal winds. Figure 5A and B shows the wind shear strength and direction during the eclipse day. As the eclipse time is during 14–17 UTC, we showed the wind shear during 12 UTC and 18 UTC using the 6-hourly ERA5 data. This data is used from European Centre for Medium‐Range Weather Forecasts (ECMWF) Reanalysis 5 product *ECMWF*^32^, which can be downloaded from the following link (https://cds.climate.copernicus.eu/). During the eclipse totality region, there is a strong wind shear exists between the mesosphere and the stratosphere. The eclipse totality at 350 km altitude is also indicated by a “solid line”. It suggests that the easterlies are gradually increasing from the stratosphere to the mesospheric altitudes in the eclipse central region. While the north and south of eclipse totality, there is no much change in the wind shear strength. Hence, the north–south contrast might not be prominently associated with vertical wind shear. The other cause for the contrasting strength in the AGWs amplitude in the north and the south of the eclipse totality might be related to the meridional shear of horizontal winds.

Several studies in the past discussed the role of AGWs filtering due to background winds i.e. directional filtering of gravity waves^33^. The effect of vertically sheared winds are well known, but the effect of varying horizontal winds on the AGWs propagation least understood^30^. Simulation studies noted that the horizontal reflection of AGWs propagating through the meriodnally sheared zonal winds^34^. If the meridional sheared winds are not strong beyond the critical reflection point, the AGWs will transmit partially. The waves energy will reduce and finally evanescent when propagating direction is similar to prevailing background wind direction as they deliver their momentum to the background wind flow. While waves moving in opposite direction gain momentum from the background wind. Further, it is also noted that the wave moving perpendicular to the wind are unaffected by the flow^30,35^. Hence, we further show the background flow from the ERA5 reanalysis in the mesospheric altitudes. This is the highest altitude from the reanalysis products, which will give more realistic atmospheric winds. Figure 5C and D show the spatial distribution of wind magnitude and direction ~ 80 km. It is important to note that from the wind direction in the north and south of the eclipse totality region. On the northern side of eclipse totality, we could see strong south-easterlies, while on the southern side the easterlies prevail between 12 and 18UTC. This indicates there is change in the prevailing wind direction to the north and south of the eclipse path. Hence, there is a higher probability of northward propagation of AGWs relative to the south. This is because the propagation direction of AGWs can be more or less related to the bow waves like the ship wakes^36^. This wave direction considering the eclipse path shown in Fig. 5A and B might be perpendicular to the background wind direction. Hence, we here speculate that the wave direction is more or less perpendicular to the north side than the southern side and the waves are relatively less affected while they propagate in the northern direction and along the eclipse path and supporting present observations of relatively high amplitude AGWs at the site north of totality and for PRN29. Nevertheless, more detailed modelling studies are needed to further confirm the role of wind direction and eclipse generated AGWs. This will further support our arguments discussing north–south differences in the AGWs amplitude from the eclipse path.

### Comparison of effects of TSE2019 and TSE2020 on the ionospheric plasma desnity

**Figure 6:**
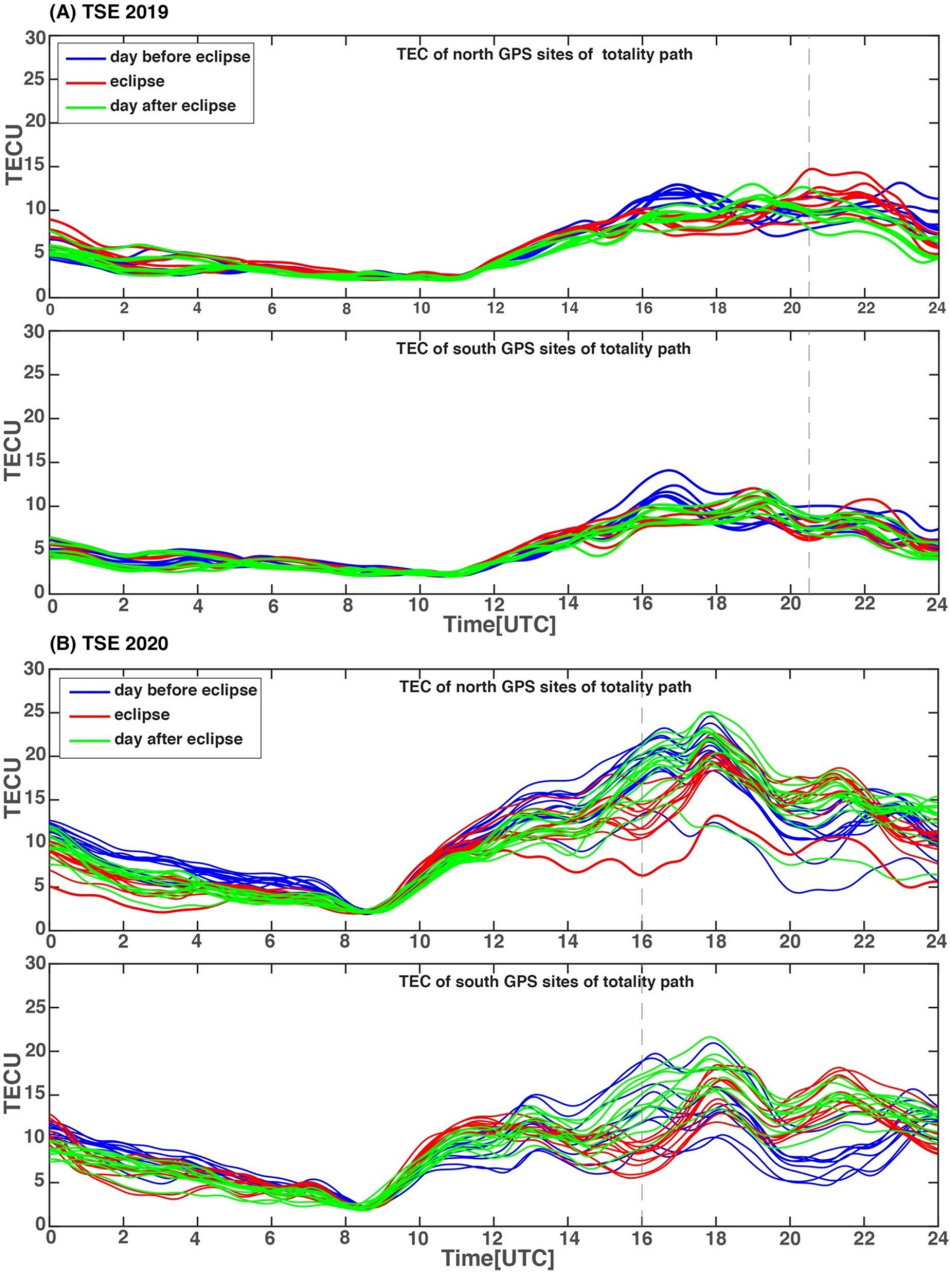
(**A**, **B**) Show daily VTEC variation for TSE2019 and TSE2020 for three days, day before eclipse (blue color lines), eclipse day (red color) and day after eclipse (green color). The upper panel showing VTEC variation for sites located north of totality line while lower panel show VTEC variation for sites located south of totality line. The Figure is prepared using the GMT 5.4.5^37^.

The two solar eclipses (TSE2019 and TSE2020) occurred at a nearly similar geographic location and geomagnetic conditions, thus, provide a unique opportunity for comparison of their effect on the ionospheric plasma density. The major observations of TSE2019, was the peculiar feature in VTEC variation which showed an increase in VTEC on eclipse day compare to a normal day for the GPS sites located north of central totality lines. The sites located south of the totality line, showed a normal eclipse effect, i.e., a decrease in VTEC on eclipse day compared to a normal day. In the present case, the VTEC on eclipse day shows normal variation i.e., a decrease in VTEC values compare to the mean value at the GPS sites located on both sides of the totality path. The background wind analysis results for TSE2020 suggest south-easterly wind prevailed during eclipse time, while during TSE2019, the south-westerlies were found prominent. Thus, background wind direction during both eclipse time was supporting the AGWs propagation towards northward. Though we do observe stronger amplitude AGWs for the sites located north of the totality line during TSE2020, it does not translate in to increase in electron density on eclipse day as compare to during TSE2019. To better understand this difference, we have analyzed background VTEC during both eclipses. The background TEC is the average (2 sigma iterated) TEC over all the PRNs for 24 h duration after adding satellite and receiver biases. This is calculated for each site and provides overall TEC variation at the given sites. The background TEC is estimated using GPS_GOPI software (http://seemala.blogspot.com/2017/09/gps-tec-program-ver-295.html) and is contained in the “.Std” output files. Figure 6A and B show VTEC variation for both eclipses during the day before the eclipse (blue colour lines), eclipse day (red colour) and the day after the eclipse day (green colour). The upper panel show the GPS sites north of the totality lines and the lower panel for the GPS sites south of the totality line respectively in both Fig. 6A and B. For TSE2019 (Fig. 6A, upper panel) the VTEC diurnal variation on the day before the eclipse (blue plots) show peak ~ 17UT, on the eclipse day (red plots) peaked after ~ 21 UT, and for the day after the eclipse (green plots) peak observed at ~ 19UT, thus there is a large day to day variability, on three days. The sites at the south of totality (Fig. 6A, lower panel) show lesser day to day variability. For TSE2020, (Fig. 6B, upper and lower panel) TEC on three days peaked ~ 18 UT, thus, show fewer day to day variability and a more pronounced eclipse effect (decrease in VTEC) observed. Hence, large day to day variability in background electron density may also play an important role in determining resultant electron density during the eclipse apart from the AGWs. Here, we should also note the basic difference during two eclipses, local time, season (TSE2019 occurred during the late afternoon to evening in the winter while TSE2020 occurred early forenoon to afternoon time during summer season), geographic location and eclipse geometry (TSE2019 occurred more northward and were close to the end of totality path, while TSE2020 occurred more southward and was close to maximum eclipse). Another important noteworthy difference is the background wind direction which was westerly for TSE2019, while it was easterly during TSE2020. Due to these noteworthy differences, it is always challenging to compare the observational results of two eclipses. Despite these limitations, our results indicate an important role of eclipse generated AGWs and background electron density.

## Summary

The TSE2020 occurred on 14th December 2020 over the South American region. The totality path with eclipse magnitude 1.012 passed through the Villarrica south Chile 14:41:02.0 UTC (10:41:07.9 CLT) to 17:30:58.1 UTC (13:30:58.1 CLT) and maximum 16:03:49.5 UTC (12:03:49.5 CLT). The effect of this eclipse on the ionospheric is studied by using the continuous Chilean Global Positioning System (GPS) sites across the totality path which includes ~ 36 sites located across both sides of the totality line. The two most suitable PRN’s 29 and 31 were selected to derive the VTEC values at each site. The VTEC values show almost 20–40% of reduction with reference to ambient values. The reduction was maximum close to totality site and decreases smoothly on both side of totality sites. Interestingly, AGWs with a period ~ 30–60 min obtained using wavelet analysis of VTEC time-series show the presence of strong AGWs at the GPS sites located north of the totality line. But the AGWs do not show any significant effect on the VTEC values to these sites. We further compared the observational results of two eclipses TSE2019 and TSE2020 due to their similar geographic locations and found the presence of the large day to day variability in the background VTEC values during TSE2020. Thus, it is possible that due to the presence of large variability in the background plasma density, and eclipse generated AGWs induced plasma density perturbation balance each other and only eclipse induced reduction in the ionization rate is seen as a smooth reduction in the plasma density during the eclipse. Therfore we propose a more detailed study by taking many cases of eclipse event with similar conditions supported by modelling work to further confirm these finding and to reach a conclusion.

## Supplementary Information

Supplementary Information.

## References

[1] Antonia RA, Chambers AJ, Phong-Anant D, Rajagopalan S, Sreenivasan KR (1979). Response of atmospheric surface layer turbulence to a partial solar eclipse. J. Geophys. Res..

[2] Chimonas G, Hines CO (1970). Atmospheric gravity waves induced by a solar eclipse. J. Geophys. Res..

[3] Chimonas G (1970). Internal gravity-wave motions induced in the Earth’s atmosphere by a solar eclipse. J. Geophys. Res..

[4] Maurya AK, Phanikumar DV, Singh R, Kumar S, Veenadhari B, Kwak YS, Niranjan Kumar K (2014). Low-mid latitude D region ionospheric perturbations associated with 22 July 2009 total solar eclipse: Wave-like signatures inferred from VLF observations. J. Geophys. Res. Space Physics.

[5] Phanikumar DV, Kwak YS, Patra AK, Maurya AK, Singh R, Park SM (2014). Response of the mid-latitude D-region ionosphere to the total solar eclipse of 22 July 2009 studied using VLF signals in South Korean peninsula. Adv. Space Res..

[6] Singh R, Veenadhari B, Maurya AK, Cohen MB, Kumar S, Selvakumaran R, Inan US (2011). D-region ionosphere response to the total solar eclipse of 22 July 2009 deduced from ELF-VLF tweek observations in the Indian sector. J. Geophys. Res..

[7] Rishbeth H (1968). Solar eclipses and ionospheric theory. Space Sci. Rev..

[8] Rishbeth H (1970). Eclipse effects in the ionosphere. Nature.

[9] Hines CO (1960). Internal atmospheric gravity waves at ionospheric heights. Can. J. Phys..

[10] Fritts DC, Luo Z (1993). Gravity wave forcing in the middle atmosphere due to reduced ozone heating during a solar eclipse. J. Geophys. Res..

[11] Liu JY, Hsiao CC, Tsai LC, Liu CH, Kuo FS, Lue HY, Huang CM (1998). Vertical phase and group velocities of internalgravity waves derived from ionograms during the solareclipse of 24 October 1995. J. Atmos. Solar Terr. Phys..

[12] Maurya AK, Shrivastava MN, Kumar KN (2020). Ionospheric monitoring with the Chilean GPS eyeball during the South American total solar eclipse on 2nd July 2019. Sci. Rep..

[13] Vadas SL, Fritts DC, Alexander MJ (2003). Mechanism for the generation of secondary waves in wave breaking regions. J. Atmos. Sci..

[14] Tsai HF, Liu JY (1999). Ionospheric total electron content response to solar eclipses. J. Geophys. Res. Space Phys..

[15] Afraimovich EL, Palamartchouk KS, Perevalova NP, Chernukhov VV, Lukhnev AV, Zalutsky VT (1998). Ionospheric effects of the solar eclipse of March 9, 1997, as deduced from GPS data. Geophys. Res. Lett..

[16] Vyas BM, Sunda S (2012). The solar eclipse and its associated ionospheric TEC variations over Indian stations on January 15, 2010. Adv. Space Res..

[17] Kumar KV, Maurya AK, Kumar S, Singh R (2016). 22 July 2009 total solar eclipse induced gravity waves in ionosphere as inferred from GPS observations over EIA. Adv. Space Res..

[18] Nayak C, Yiğit E (2018). GPS-TEC observation of gravity waves generated in the ionosphere during 21 August 2017 total solar eclipse. J. Geophys. Res. Space Phys..

[19] Jonah OF, Goncharenko L, Erickson PJ, Zhang S, Coster A, Chau JL, Paula ERD, Rideout W (2020). Anomalous behavior of the equatorial ionization anomaly during the 2 July 2019 solar eclipse. J. Geophys. Research Space Phys..

[20] Hernández-Pajares M, Juan JM, Sanz J (2006). Medium-scale traveling ionospheric disturbances affecting GPS measurements: Spatial and temporal analysis. J. Geophys. Res. Space Phys..

[21] Verhulst TG, Stankov SM (2020). Height dependency of solar eclipse effects: The ionospheric perspective. J. Geophys. Res: Space Phys..

[22] Rao, P. R., Krishna, S. G., Niranjan, K., & Prasad, D. S. V. V. D. (2006). Temporal and spatial variations in TEC using simultaneous measurements from the Indian GPS network of receivers during the low solar activity period of 2004? 2005.

[23] Seemala GK, Valladares CE (2011). Statistics of total electron content depletions observed over the South American continent for the year 2008. Radio Sci..

[24] Schaer, S. & Société helvétique des sciences naturelles. Commission géodésique. *Mapping and Predicting the Earth's Ionosphere Using the Global Positioning System* (Vol. 59). (Institut für Geodäsie und Photogrammetrie, 1999)

[25] Rastogi RG, Sharma RP (1971). Ionospheric electron content at Ahmedabad (near the crest of equatorial anomaly) by using beacon satellite transmissions during half a solar cycle. Planet. Space Sci..

[26] Rao, P. V. S., Rao, M. S., & Satyam, M. (1977). D*iurnal & Seasonal Trends in TEC Values Observed at Waltair*.

[27] Grossmann A, Morlet J (1984). Decomposition of Hardy functions into square integrable wavelets of constant shape. SIAM J. Math. Anal..

[28] Šauli P, Abry P, Boška J, Duchayne L (2006). Wavelet characterisation of ionospheric acoustic and gravity waves occurring during the solar eclipse of August 11, 1999. J. Atmos. Solar Terr. Phys..

[29] Somsikov VM, Ganguly B (1995). On the formation of atmospheric inhomogeneities in the solar terminator region. J. Atmos. Terr. Phys..

[30] Heale CJ, Snively JB (2015). Gravity wave propagation through a vertically and horizontally inhomogeneous background wind. J. Geophys. Res..

[31] Hysell D, Larsen M, Fritts D, Laughman B, Sulzer M (2018). Major upwelling and overturning in the mid-latitude F region ionosphere. Nat. Commun..

[32] European Centre for Medium‐Range Weather Forecasts. Updated monthly. ERA5 Reanalysis. Research Data Archive at the National Center for Atmospheric Research, Computational and Information Systems Laboratory. 10.5065/D6X34W69 (2017).

[33] Hines CO, Reddy CA (1967). On the propagation of atmospheric gravity waves through regions of wind shear. J. Geophys. Res..

[34] Huang KM, Zhang SD, Yi F (2008). Propagation and reflection of gravity waves in a meridionally sheared wind field. J. Geophys. Res. Atmospheres.

[35] Talaei, A. (2016). On the propagation of atmospheric gravity waves in a non-uniform wind field: Introducing a modified acoustic-gravity wave equation.

[36] Lin CY, Deng Y, Ridley A (2018). Atmospheric gravity waves in the ionosphere and thermosphere during the 2017 solar eclipse. Geophys. Res. Lett..

[37] Wessel P, Smith WHF, Scharroo R, Luis J, Wobbe F (2013). Generic mapping tools: improved version released. Trans. Am. Geophys. Union.

