# Supplementary material for: Ionospheric perturbation during the South American total solar eclipse on 14th December 2020 revealed with the Chilean GPS eyeball
Source: Sci Rep. 2021 Oct 13;11:20324. doi: 10.1038/s41598-021-98727-w (PMC8514497; doi:10.1038/s41598-021-98727-w)
Supplement: Supplementary file 1 — Supplementary Information. [file 41598_2021_98727_MOESM1_ESM.docx]

**Ionospheric effects of South American total solar eclipse of 14^th^ December 2020 revealed with the Chilean GPS eyeball**

Mahesh N Shrivastava^1^, Ajeet K Maurya^2*^ and Kondapalli Niranjan Kumar^3^

^1^ Universidad Católica del Norte, Antofagasta, Chile

^2^ Department of Physics, Doon University, Dehradun, India

^3^ National Centre for Medium Range Weather Forecasting, Ministry of Earth Sciences, India.

*Corresponding author: [ajeet.iig@gmail.com](mailto:ajeet.iig@gmail.com)

**Supplementary Information**

**Figures:** S1 - S4

**Table:** 1

**
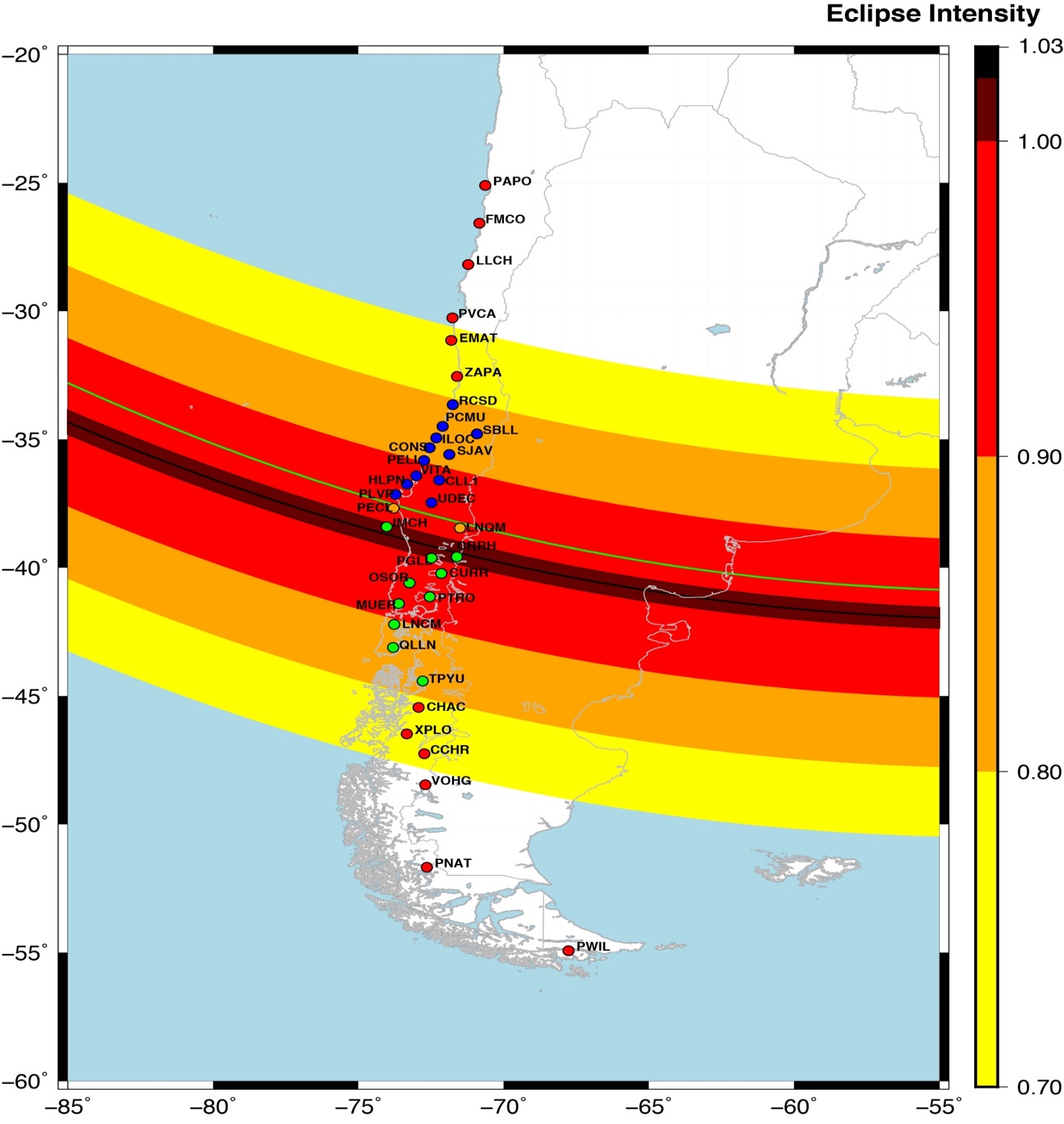
**

**Figure S1:** The path of the total solar eclipse showing in the Chilean region. The dots show the GPS sites. Red dots GPS sites are the out of 80% totality region, blue and green in the 80% totality region in the north and south respectively. The Figure is prepared using the GMT 5.4.5^36^.

**
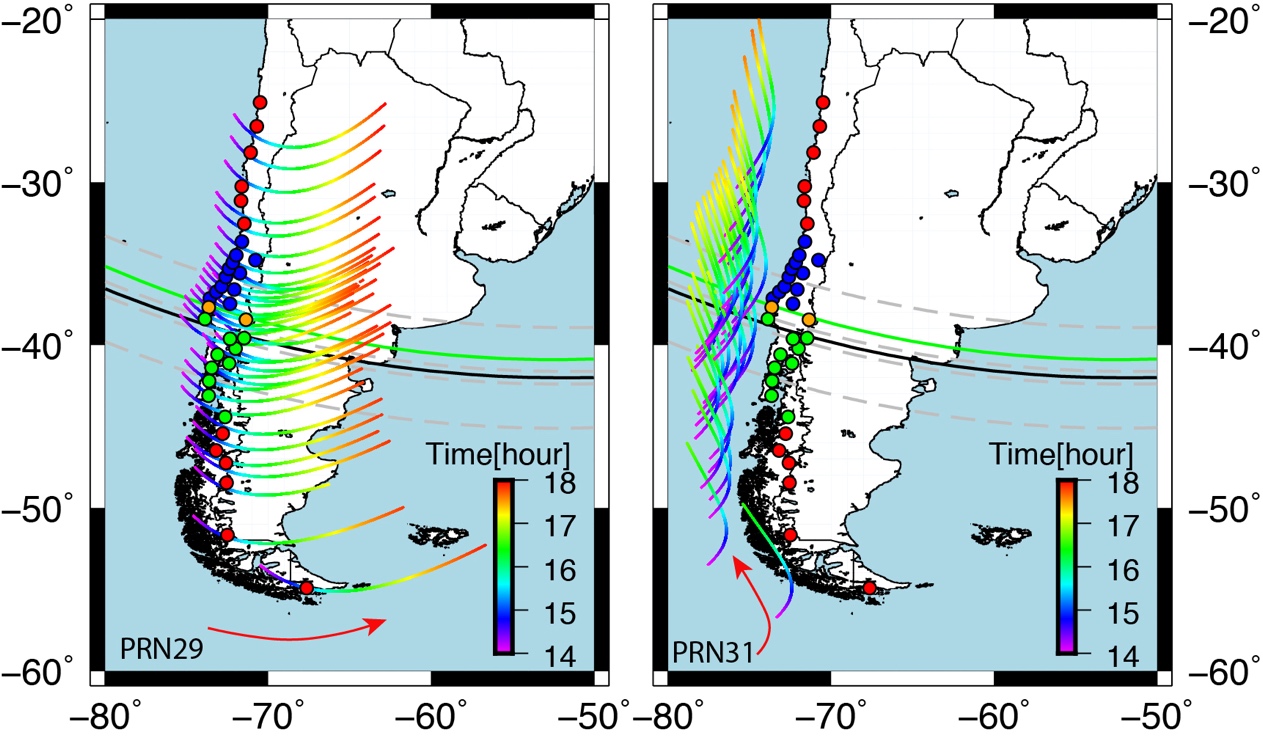
**

**Figure S2:** In the left panel, the UTC time of GPS PRN29 path at 350 km altitude with respect to ground GPS satellites with color table from blue to red and IPP represent on the path with color circles. In the right panel. the UTC time of GPS PRN31 path at 350 km altitude with respect to ground GPS satellites with color table from blue to red and IPP represent on the path with color circles. The Figure is prepared using the GMT 5.4.5^36^.


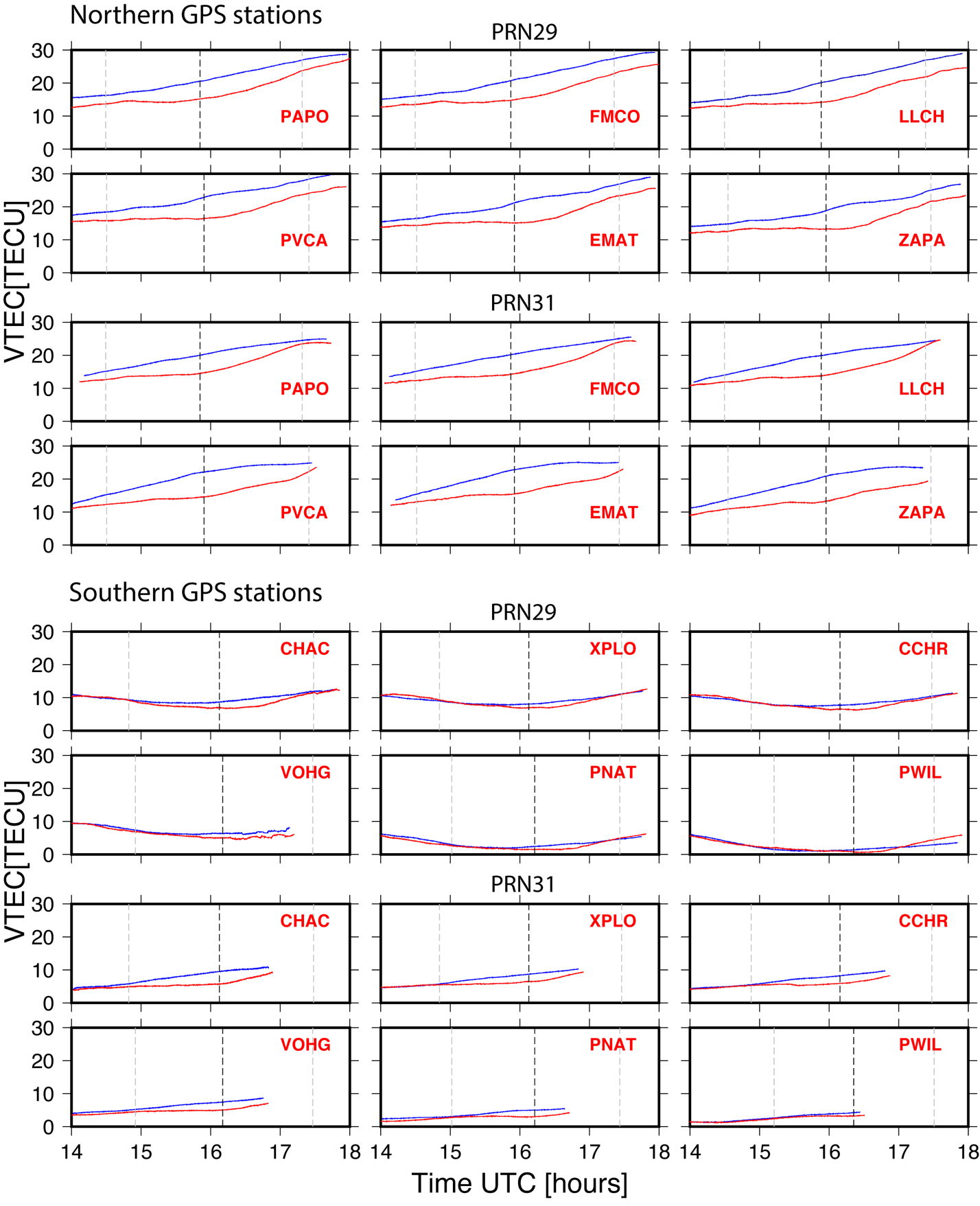


**Figure S3:** VTEC plots of PRN 29 and PRN 31 of the northern and southern GPS stations away from 80% totality region. The Figure is prepared using the GMT 5.4.5^36^.


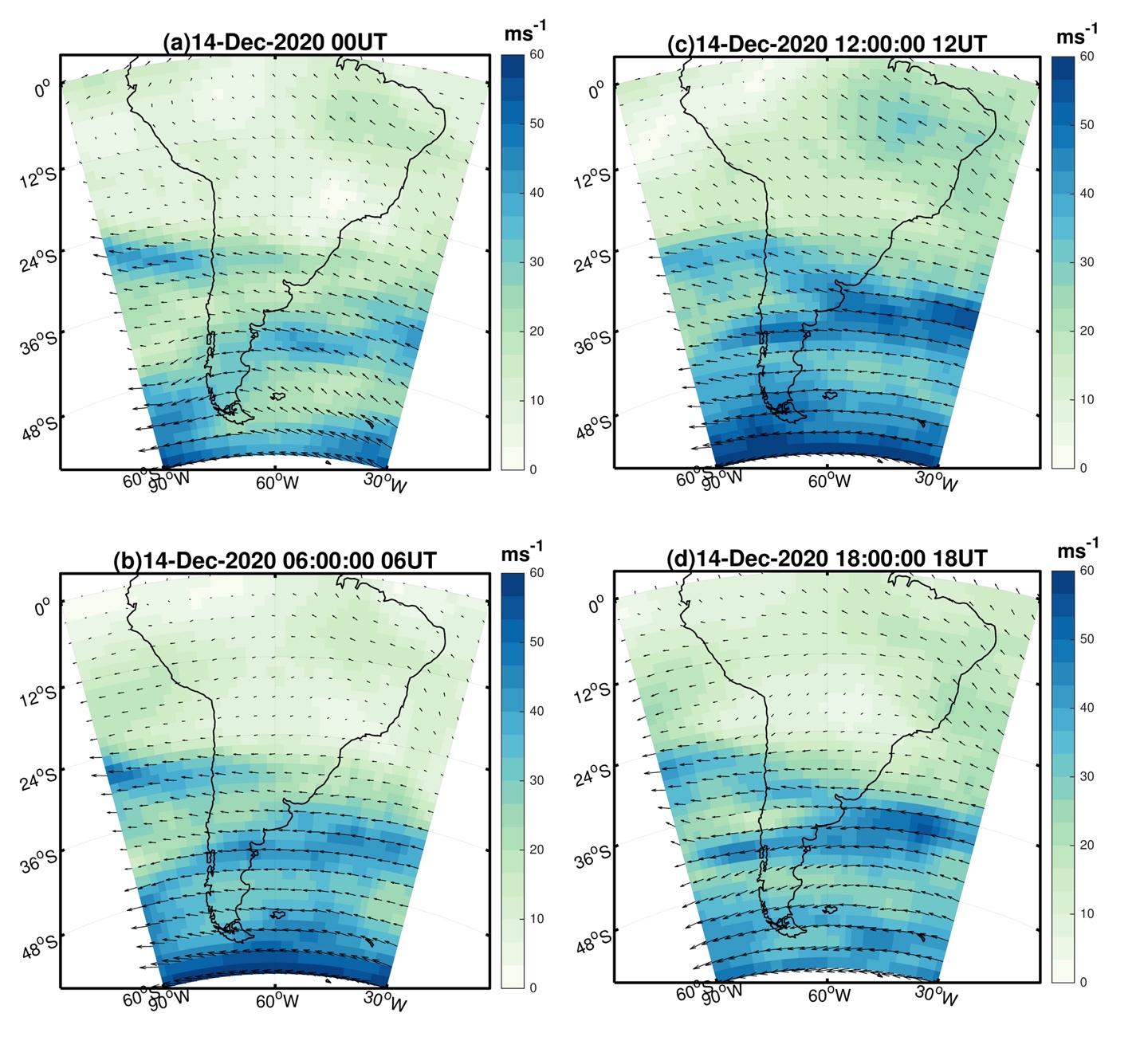


**Figure S4:** The background wind speed (shaded) and vector winds over the South America on for four different timings (0, 6, 12 and 18 UTC) on Total Solar Eclipse Day on Dec 14, 2020, at ~ 80 km from ERA5 reanalysis data. The Figure is prepared using the GMT 5.4.5^36^.

**Table S1:** List of GPS stations of northern and southern of the total solar eclipse.

| **S/N** | **GPS**  **station** | **Lat[^o^]** | **Long[^o^]** | **Eclipse**  **start time**  **[UTC]** | **Max**  **eclipse**  **time [UTC]** | **Eclipse**  **end time**  **[UTC]** | **%Obscuration**  **(Magnitude)** | **VTEC**  **PRN29** | | **VTEC**  **PRN31** | |
| --- | --- | --- | --- | --- | --- | --- | --- | --- | --- | --- | --- |
|  |  |  |  |  |  |  |  | **Time** | **Max** | **Time** | **Max** |
| 1 | PAPO | -25.10 | -70.49 | 14.492 | 15.848 | 17.316 | 47.74(0.573) | 16.082 | -5.835 | 16.002 | -5.935 |
| 2 | FMCO | -26.57 | -70.69 | 14.495 | 15.870 | 17.355 | 53.20(0.620) | 16.235 | -6.390 | 15.985 | -6.260 |
| 3 | LLCH | -28.19 | -71.08 | 14.496 | 15.886 | 17.385 | 59.58(0.673) | 16.138 | -6.340 | 15.945 | -6.380 |
| 4 | PVCA | -30.29 | -71.62 | 14.505 | 15.905 | 17.412 | 67.90(0.741) | 16.195 | -7.090 | 15.969 | -7.600 |
| 5 | EMAT | -31.26 | -71.66 | 14.517 | 15.921 | 17.428 | 71.22(0.767) | 16.203 | -7.140 | 15.983 | -7.630 |
| 6 | ZAPA | -32.55 | -71.46 | 14.547 | 15.956 | 17.462 | 76.25(0.807) | 16.307 | -7.355 | 16.009 | -7.705 |
| 7 | CHAC | -45.45 | -72.78 | 14.823 | 16.126 | 17.480 | 79.08(0.830) | 16.695 | -2.545 | 16.222 | -3.820 |
| 8 | XPLO | -46.48 | -73.19 | 14.845 | 16.129 | 17.462 | 75.05(0.798) | 16.561 | -1.635 | 16.297 | -2.460 |
| 9 | CCHR | -47.25 | -72.59 | 14.880 | 16.156 | 17.476 | 72.86(0.781) | 16.424 | -1.735 | 16.283 | -2.505 |
| 10 | VOHG | -48.46 | -72.55 | 14.918 | 16.173 | 17.468 | 68.66(0.747) | 17.070 | -2.960 | 16.220 | -2.535 |
| 11 | PNAT | -51.67 | -72.50 | 15.021 | 16.213 | 17.436 | 57.88(0.659) | 16.591 | -1.525 | 16.155 | -2.040 |
| 12 | PWIL | -54.93 | -67.63 | 15.209 | 16.353 | 17.508 | 50.09(0.594) | 16.582 | -1.050 | 16.383 | -1.110 |
